# Probiotic Administration Increases Amino Acid Absorption from Plant Protein: a Placebo-Controlled, Randomized, Double-Blind, Multicenter, Crossover Study

**DOI:** 10.1007/s12602-020-09656-5

**Published:** 2020-05-01

**Authors:** Ralf Jäger, Javier Zaragoza, Martin Purpura, Stefania Iametti, Mauro Marengo, Grant M. Tinsley, Anthony J. Anzalone, Jonathan M. Oliver, Walter Fiore, Andrea Biffi, Stacie Urbina, Lem Taylor

**Affiliations:** 1 Increnovo, Milwaukee, WI USA; 2 Human Performance Laboratory, School of Exercise & Sport Science, University of Mary Hardin-Baylor, Belton, TX USA; 3 Department of Food, Environmental and Nutritional Sciences (DeFENS), Università degli Studi di Milano, Milan, Italy; 4 Energy Balance & Body Composition Laboratory, Texas Tech University, Lubbock, TX USA; 5 Wake Forest School of Medicine, Winston-Salem, NC USA; 6 Army West Point Athletics Association, West Point, NY USA; 7 Sofar S.p.A., Trezzano Rosa, Italy

**Keywords:** Protein, Probiotic, Amino acid absorption, Muscle health

## Abstract

The fate of dietary protein in the gut is determined by microbial and host digestion and utilization. Fermentation of proteins generates bioactive molecules that have wide-ranging health effects on the host. The type of protein can affect amino acid absorption, with animal proteins generally being more efficiently absorbed compared with plant proteins. In contrast to animal proteins, most plant proteins, such as pea protein, are incomplete proteins. Pea protein is low in methionine and contains lower amounts of branched-chain amino acids (BCAAs), which play a crucial role in muscle health. We hypothesized that probiotic supplementation results in favorable changes in the gut microbiota, aiding the absorption of amino acids from plant proteins by the host. Fifteen physically active men (24.2 ± 5.0 years; 85.3 ± 12.9 kg; 178.0 ± 7.6 cm; 16.7 ± 5.8% body fat) co-ingested 20 g of pea protein with either AminoAlta™, a multi-strain probiotic (5 billion CFU *L. paracasei* LP-DG® (CNCM I-1572) plus 5 billion CFU *L. paracasei* LPC-S01 (DSM 26760), SOFAR S.p.A., Italy) or a placebo for 2 weeks in a randomized, double-blind, crossover design, separated by a 4-week washout period. Blood samples were taken at baseline and at 30-, 60-, 120-, and 180-min post-ingestion and analyzed for amino acid content. Probiotic administration significantly increased methionine, histidine, valine, leucine, isoleucine, tyrosine, total BCAA, and total EAA maximum concentrations (Cmax) and AUC without significantly changing the time to reach maximum concentrations. Probiotic supplementation can be an important nutritional strategy to improve post-prandial changes in blood amino acids and to overcome compositional shortcomings of plant proteins. ClinicalTrials.gov Identifier: ISRCTN38903788

## Introduction

*Lactobacillus paracasei* strains have been isolated from the intestinal and reproductive tracts of humans and animals and numerous food products such as raw milk, plants, or fermented foods [1]. There are currently no clinical trials on the combination of *L. paracasei* LP-DG® and *L. paracasei* LPC-S01; however, the individual strains have been studied for various health applications. Probiotic *L. paracasei* LP-DG® (CNCM I-1572) can survive gastrointestinal transit in children [2] and healthy adults [3] and has been shown to be able to modulate gut microbiota structure/function and immune health in healthy adults [4], patients with irritable bowel syndrome (IBS) [5, 6], and diverticular disease [7–10]. Similar to *L. paracasei* LP-DG® (CNCM I-1572), *L. paracasei* LPC-S01 (DSM 26760) has been shown to colonize the human gut of healthy adults [11]. Through optimizing gut microbiota composition, probiotics have been linked to improve nutrient absorption, including protein utilization [12].

Different types and quality of dietary protein can affect amino acid absorption following protein supplementation. Compared with animal protein sources, plant protein sources, with the exception of soy protein, are incomplete proteins lacking in one or more essential amino acids. Plant proteins contain less branched-chain amino acids (BCAAs), especially leucine [13], one of the crucial amino acids for muscle health, especially the activation of muscle protein synthesis (MPS) [14]. In addition, plant proteins differ in absorption kinetics and the amount of amino acids absorbed by the host. Therefore, there is an interest in nutritional strategies to raise the blood amino acid concentrations after ingesting a plant protein source to overcome compositional shortcomings.

The fate of dietary protein in the gut is determined by microbial and host digestion and utilization. Dietary protein is cleaved into polypeptides by proteases at low pH in the stomach. Further degradation in the intestine by luminal proteases and membrane bound peptidases results in the formation of peptides and amino acids. Amino acids enter the cell via various transmembrane transport proteins, amino acid transporters, while di- and tripeptides are absorbed into the intestinal cells via the mammalian proton-coupled peptide transporter PEPT1. In the cytosol, peptides are mostly degraded leaving the cell as amino acids. Bacterial proteolytic fermentation in the gut competes with host digestion, significantly contributing to the metabolite pool in the large intestine and contributing to host amino acid balance in the small intestine. Microbial protein fermentation alters the gut microbiota composition and generates a diverse range of bioactive molecules which exert wide-ranging host effects [15].

While lactic acid bacteria (LAB) have proteases and peptidases that provide the bacteria with free amino acids for optimal growth, they are not classified as strongly proteolytic bacteria. The role of LAB such as *L. paracasei* on protein digestion and absorption of amino acids by the host is currently unknown.

The purpose of this study was to investigate the individual and potential additive or synergistic effects of *L. paracasei* LP-DG® (CNCM I-1572) and/or *L. paracasei* LPC-S01 (DSM 26760) on the in vitro digestion of different plant protein sources, followed by a human study investigating blood amino acid concentrations after co-administration of plant protein and probiotics.

## Materials and Methods

### In Vitro Tests

In vitro proteolytic effects of the two probiotic strains towards different plant protein sources have been investigated by incubating suspensions of either pea or rice protein isolates with the probiotic bacterial strains, in the presence and in the absence of porcine pepsin (EC 3.4.23.1; Sigma-Aldrich, Milan, Italy) and pancreatin (Sigma-Aldrich, Milan, Italy). In particular, 0.8 mL of protein isolate suspension (10 g/L) was mixed with 0.02 mL of porcine gastric pepsin solution (2 g/L in water). After 1-h incubation at 37 °C under mixing, the pH was adjusted to ≈ 8.0 by adding 1 M Tris base, and 0.2 mL pancreatin from porcine pancreas (2 g/L in 0.1 M Tris-HCl buffer, pH 7.5; EC 232-468-9) were added. Pancreatin digestion was carried out for 3 h in the absence or in the presence (when appropriate) of 0.1 mL of probiotic cells (250 g/L in 0.1 M Tris-HCl buffer, pH 7.5). To address the proteolytic activity of the bacteria alone on the various substrates, reaction mixtures were prepared by using the probiotic strains either individually or in combination, but without adding any proteases. Also, as a negative control, 0.1 mL bacterial cell suspensions were mixed with 0.1 M Tris-HCl buffer, pH 7.5.

Peptides released upon proteolysis in all tested conditions were assessed by adding to an appropriate aliquot of each reaction mixture trichloroacetic acid (TCA) up to a final 10% amount. After centrifuging at 10000×*g* for 25 min at room temperature, the absorbance at 280 nm of the supernatant provides the amount of soluble peptides.

For SDS-PAGE analysis, adequate amounts of the different reaction mixtures at various times of incubation were diluted (1/1, v/v) with Laemmli denaturing buffer (0.125 M Tris–HCl, pH 6.8; 50% glycerol; 1.7% SDS; 0.01% bromophenol blue; 1% 2-mercaptoethanol) and heated at 100 °C for 10 min. SDS-PAGE was carried out on a 12% monomer gel, by using a MiniProtein apparatus (Bio-Rad, Richmond, VA, USA), and gels were Coomassie Blue-stained.

Aliquots of the various reaction mixtures were treated with 0.1% trifluoroacetic acid (TFA) and subsequently centrifuged at 12000×*g* for 20 min. A 0.2 mL aliquot of the soluble material was loaded onto a Symmetry C18 column (300 Å; 3.5 μm; 2.1 mm × 50 mm; Waters, Milan, IT), previously equilibrated with 0.1% TFA in distilled water, and eluted at a flow rate of 0.8 mL/min, with a linear gradient starting 5 min after injection up to 60% (v/v) acetonitrile, in the presence of 0.1% TFA. The chromatographic separation was carried out by a Waters™ 626 system and equipped with a Waters™ 2487 dual wavelength detector (Waters, Milan, Italy). Data at 220 nm and at 280 nm were recorded and elaborated by using the software Empower Pro (Waters, Milan, Italy). Results are expressed as total peak area measured at 220 nm that include the peptides that do not contain aromatic residuals that were quantified measuring the absorbance at 280 nm of the supernatant after TFA treatment.

### Human Clinical Study

This multicenter study was conducted at Texas Christian University (TCU), Fort Worth, TX, USA, and the University of Mary Hardin-Baylor (UMHB), Belton, TX, USA, in accordance with the Declaration of Helsinki guidelines and registered with the ISRCTN registry (ISRCTN38903788). All the procedures were approved by the Institutional Review Board of Texas Christian University (IRB approval: 1707-080-1707) and University of Mary Hardin-Baylor (IRB approval: 44). Despite this being a multicenter trial, all blood analyses were sent to Heartland Assays, Iowa State University Research Park, Ames, IA, USA, to eliminate the risk of a study site effect.

#### Participants

Fifteen healthy men (TCU = 10; UMHB = 5; *n* = 15) between the ages of 18–35 years were recruited to participate in this study (Table 1). In order to qualify for this study, participants needed to have normal body weight (body mass index (BMI) of 19–24.99 kg/m^2^) and be recreationally active (according to the American College of Sports Medicine Guidelines). Subjects were not allowed to consume any nutritional or ergogenic supplement known to affect measures of the current study for the prior 6 weeks, including probiotics, prebiotics, and digestive enzymes. Exclusion criteria included any individual who was treated for or diagnosed with a gastrointestinal, cardiac, respiratory, circulatory, musculoskeletal, metabolic, immune, autoimmune, psychiatric, hematological, neurological, or endocrinological disorder. Participants who were determined to not be weight stable defined as measured body mass deviating by 2% or more, and participants who were not willing to abstain from alcohol, nicotine, and caffeine for 12 h prior to each visit were excluded. Participants who met the necessary inclusion criteria were further encouraged not to change their current physical activity levels and to refrain from exercise for 24 h before starting the clinical trial. At TCU, participants were randomized via the RAND function in Microsoft Excel. Of the 10 participants completed at TCU, 3 completed the treatment first, while the other 7 completed the placebo. At UMHB, participants were randomized using Random.org. Of the 5 participants completed at UMHB, all completed the treatment first and placebo second.

**Table 1:** Subject characteristics at baseline (*n* = 15)

Age (years)	24.2 ± 5.0
Height (cm)	178.0 ± 7.6
Weight (kg)	85.3 ± 12.9
Body Fat (%)	16.7 ± 5.8

Data is presented as means ± standard deviation

#### Experimental Protocol

A randomized, double-blind, crossover pilot study was performed to assess the amino acid concentration in the blood after the administration of a plant protein with or without co-administration of a probiotic supplement. Prior to beginning the study, all participants signed an IRB-approved informed consent document and completed a health history questionnaire to determine study eligibility. Two supplementation periods that each spanned 2 weeks were completed and separated by a washout period of 4 weeks. For each study visit, all participants reported to the laboratory between 06:00 and 10:00 h after observing an 8–10-h fast. Daily diet was recorded, and subjects were asked to repeat the same diet for 2 weeks leading up to the second experimental testing. Participants were randomly assigned to ingest either a single daily 20-g dose of a pea protein plus placebo or a single daily 20-g dose of a pea protein plus probiotic. Upon arrival for each study visit, participants had their resting heart rate, blood pressure, body mass, height, and body fat measured using skinfold. The occurrence of adverse events was recorded throughout completion of the supplementation and study visits. Adverse events were collected through spontaneous reporting by the study participants, clinical evaluation, or interaction of a research team member with a study participant. Subjects rested semi-supine for placement of a Teflon catheter into an antecubital vein for multiple blood sampling. The catheter was kept patent by flushing with 3 ml of 0.9% sodium chloride. Following baseline sampling, participants ingested their respective supplement. Thereafter, blood samples were taken at 30-, 60-, 120-, and 180-min post-ingestion (Table 2). Subsequently, a 4-week washout period was implemented, followed by the opposite condition. Whole blood was collected and transferred into Becton Dickinson (BD) 8.5 ml tubes (BD Vacutainer SST) for obtaining serum and BD 10.0 ml (Vacutainer Sodium Heparin) tubes for obtaining plasma and subsequently centrifuged at 1500×*g* for 15 min at 4 °C. Resulting serum and plasma were then aliquoted and stored at − 80 °C until subsequent analyses.

**Table 2:** Study design

Screening and consent	Baseline testing	Day 1–14	Day 15	Day 16–43	Day 44–57	Day 58
Screening	Body height and weight	2 weeks of supplementation + dietary recording	Body height and weight	4 weeks of washout	2 weeks of supplementation + dietary recording	Body height and weight
Informed consent	Vitals		Supplementation adherence check			Supplementation adherence check
Demographics	Instructions for supplementation		Dietary recording check			Dietary recording check
Health history	Instructions for dietary recording		Adverse event check			Adverse event check
Exercise history	Dietary restrictions		Fasting blood draw			Fasting blood draw
Body height and weight			Ingestion of plant protein with or without a probiotic			Ingestion of plant protein with or without a probiotic
Vitals			Subsequent blood draws (30, 60, 120, 180 min)			Subsequent blood draws (30, 60, 120, 180 min)
Randomization						

#### Supplementation

Participants co-administered 20 g of protein (vegetable protein isolated from yellow pea (*Pisum sativum*), NUTRALYS® S85F, Roquette Freres S.A., France) with either 10 billion CFU of a multi-strain probiotic (5 billion CFU *L. paracasei* LP-DG® (CNCM I-1572) and 5 billion CFU *L. paracasei* LPC-S01 (DSM 26760), AminoAlta™, SOFAR S.p.A., Italy) or a placebo (maltodextrin, Glucidex® 12, Roquette Freres S.A., France), in a randomized order. The study materials were provided in a dual chamber sachet, separating the protein from the probiotic or placebo until the time of consumption. Subjects were instructed to open the pouch, mix the ingredients with 473 ml of their favorite non-protein containing beverage, and consume the product immediately. On weekdays, subjects reported to the lab daily and consumed the study materials in front of the researchers to ensure compliance. On Fridays, subjects received two additional sachets to be taken during the weekend. Study material identity and potency was verified by an independent lab (for probiotic, Covance Laboratories Inc., Madison, WI, USA; for protein content and amino acid composition, Table 3, Eurofins, Madison, WI, USA) after the completion of the study and blinded analysis of the results.

**Table 3:** Amino acid profile of the study material (Eurofins Certificate of Analysis, 2,696,852–0, November 20, 2019)

Amino acid	[mg/g of protein]
Alanine	43
Arginine	88
Aspartic acid	117
Cysteine	9
Glutamic acid	167
Glycine	41
Histidine	25
Isoleucine	48
Leucine	83
Lysine	74
Methionine	12
Phenylalanine	54
Proline	45
Serine	50
Threonine	40
Tryptophan	10
Tyrosine	39
Valine	51

#### Amino Acid Analysis

EZ:faast® amino acid analysis kits (Phenomenex, Torrance, CA) were used for liquid chromatographic analysis of amino acids using tandem-mass spectrometry (LC/MS/MS) and electrospray ionization (ESI). The procedure consisted of solid phase extraction of 25 μl of plasma with internal standards by a sorbent tip attached to a syringe with an eluting solvent (a 3:2 mixture of sodium hydroxide with 77% n-propanol and 23% 3-picoline). The free amino acids were then derivatized by adding a mixture of 17.4% propyl chloroformate, 11% isooctane, and 71.6% chloroform. The resulting mixture was vortexed and allowed to sit at room temperature for 1 min, followed by liquid-liquid extraction with isooctane. Fifty microliters of the organic layer were removed, dried under nitrogen gas, and suspended in the HPLC run solvents before being injected into the LC/MS/MS. Chromatographic separation of the derivatized amino acids was conducted on an EZ:faast amino acid analysis-mass spectrometry column (250 × 2.0 mm i.d., 4 μm) using an Agilent 6460 triple quadrupole LC/MS/MS system (Santa Clara, CA). Ten millimeters of ammonium formate in water with 0.2% formic acid (mobile phase A) and 10 mM ammonium formate in methanol with 0.2% formic acid (mobile phase B) were used as solvent system with gradient conditions of 68% B at 0 min to 83% B over 13 min with a flow rate of 0.25 ml/min. Amino acids and internal standard data were collected using the dynamic multiple reaction monitoring mode using MassHunter acquisition software (Agilent, Santa Clara, CA). MassHunter Quantitation software was used to quantitate the unknown plasma samples based on best fit standard curves.

#### Statistical Analysis

The area under the concentration vs. time curve (AUC) was calculated for each of the 22 amino acids, as well as BCAAs, EAAs, and total amino acids, via the linear trapezoidal rule and using all available time points. Cmax was defined as the maximum observed concentration, and Tmax was the time at which Cmax was reached. AUC values were compared between conditions via paired sample *t* tests. A *p* value < 0.05 was considered statistically significant. Analyses were performed in R (v. 3.6.1) using the PKNCA package. Cohen’s d effect sizes were calculated as the mean difference between conditions (i.e., protein plus probiotic minus protein) divided by the pooled standard deviation. Percent differences between treatments were calculated as the mean difference between conditions minus the value in the Protein condition times one hundred. Results are expressed as mean ± standard deviation (SD) unless otherwise noted. In vitro digestion data are the results of three independent determinations, each performed in triplicate. Analysis of variance of the in vitro digestion data was performed adopting the least significant difference (LSD). Data were analyzed using Statgraphics XV, version 15.1.02 (StatPoint, Warrenton, VA, USA).

## Results

### In Vitro Tests

SDS-PAGE analysis of the 8 different conditions showed that the combination of pepsin and pancreatin, with or without the individual or combined strains, no longer contained intact proteins (Table 4). In contrast, the individual strain alone or in combination did not show evident proteolytic activity. Analysis by RP-HPLC showed that the addition of the probiotic strains resulted in different peptides compared with digestive enzymes alone. *L. paracasei* LP-DG® showed a greater increase with rice protein in comparison with *L. paracasei* LPC-S01, whereas the combination of two strains showed a synergistic effect with pea protein. Based on the outcome of the in vitro tests, the combination of *L. paracasei* LP-DG® and *L. paracasei* LPC-S01 with pea protein was chosen for the human clinical study, to investigate if the probiotic strains can increase amino acid appearance in the blood, when co-administered with plant proteins.

**Table 4:** Analysis of overall proteolysis by RP-HPLC separation

Treatment	Rice		Pea	
	Area (*10^6^)	Δ, %	Area (*10^6^)	Δ, %
Absorbance at 220 nm				
Pepsin + pancreatin	78.2 ± 0.10		107 ± 0.60	
Pepsin + pancreatin + *L. paracasei* LP-DG	80.2 ± 0.15^*^	+ 2.6	107 ± 0.50	0
Pepsin + pancreatin + *L. paracasei* LPC-S01	78.4 ± 0.21	+ 0.3	111 ± 0.80^*^	+ 3.7
Pepsin + pancreatin + LP-DG + LPC-S01	80.6 ± 0.18^*^	+ 3.1	114 ± 0.70^**^	+ 6.5
Absorbance at 280 nm				
Pepsin + pancreatin	5.1 ± 0.10		11.9 ± 0.06	
Pepsin + pancreatin + *L. paracasei* LP-DG	5.4 ± 0.09^*^	+ 5.9	12.0 ± 0.08	+ 0.8
Pepsin + pancreatin + *L. paracasei* LPC-S01	5.2 ± 0.08	+ 2.0	12.2 ± 0.02^*^	+ 2.5
Pepsin + pancreatin + LP-DG + LPC-S01	5.4 ± 0.10^*^	+ 5.9	12.6 ± 0.07^**^	+ 5.6

Data is presented as means ± standard deviation

*Significantly different from control (*p* < 0.05)

**Significantly different from control and single strain (*p* < 0.05)

### Human Data

*L. paracasei* LP-DG® (5 billion CFU per day), *L. paracasei* LPC-S01 (5 billion CFU per day), and pea protein supplementation (20 g per day) were safe in healthy adults. No adverse events were observed during the duration of the clinical study in either group.

Probiotic administration significantly increased area under the curve (AUC) (Table 5) for methionine (+ 20.0%), histidine (+ 40.4%), valine (+ 21.5%), leucine (+ 23.3%), isoleucine (+ 26.0%), tyrosine (+ 16.0%), total BCAA (+ 22.8%), and total EAA (+ 16.0%) concentrations.

**Table 5:** Individual amino acids, total BCAA, total EAA and total amino acid area under the curve (AUC)

AUC [μmol /L • 180 min]							
Amino acid	Protein plus probiotic		Protein		Protein plus probiotic vs. protein		
	Mean	SD	Mean	SD	*p* value (*t* test)	Difference (%)	Effect size (d)
Arginine	14,490.3	4677.7	13,954.3	6438.0	0.585	3.8	0.10
Glutamine	90,876.4	21,491.3	87,569.0	25,645.0	0.649	3.8	0.14
Citrulline	3936.4	1411.6	3922.3	1906.1	0.967	0.4	0.01
Serine	17,569.1	4194.0	17,464.6	6513.0	0.942	0.6	0.02
Asparagine	13,305.5	3226.3	12,212.4	3774.1	0.337	9.0	0.31
Glycine	33,083.9	9728.7	32,626.4	10,309.8	0.874	1.4	0.05
Threonine	22,917.0	4289.4	21,905.7	6917.8	0.621	4.6	0.18
Alanine	56,904.7	13,342.8	53,750.8	20,418.2	0.496	5.9	0.18
Ornithine	10,060.4	3334.2	9793.9	3678.8	0.693	2.7	0.08
Methionine	3558.9	1462.0	2966.9	1245.0	0.007*	20.0	0.44
Proline	43,142.9	10,833.0	42,124.2	19,636.2	0.780	2.4	0.06
Lysine	36,177.2	5057.1	35,387.7	10,758.1	0.759	2.2	0.09
Aspartic acid	711.7	259.4	591.1	277.9	0.125	20.4	0.45
Histidine	14,388.0	4519.9	10,248.1	3983.4	0.009*	40.4	0.97
Valine	65,216.4	17,782.9	53,682.1	17,525.3	0.013*	21.5	0.65
Glutamic acid	6523.0	3703.0	6384.9	3079.4	0.849	2.2	0.04
Tryptophan	13,056.3	4319.6	12,078.2	3535.2	0.276	8.1	0.25
Leucine	26,443.5	10,874.2	21,447.6	10,701.1	0.006*	23.3	0.46
Phenylalanine	8879.6	2629.0	8061.8	3371.5	0.086	10.1	0.27
Isoleucine	19,048.2	6642.5	15,116.5	5961.5	0.017*	26.0	0.62
Cysteine	3016.2	932.8	3024.9	1468.0	0.978	− 0.3	− 0.01
Tyrosine	10,790.8	3532.4	9306.0	3923.5	0.009*	16.0	0.40
Total BCAA	110,886.8	33,142.0	90,304.5	33,001.5	0.008*	22.8	0.62
Total EAA	210,115.0	41,010.5	181,071.7	54,739.9	0.005*	16.0	0.60
Total amino acids	515,152.2	79,378.2	474,059.9	139,979.5	0.136	8.7	0.36

Probiotic administration significantly increased methionine (+ 16.3%), histidine (+ 49.2%), valine (+ 24.7%), leucine (+ 25.2%), isoleucine (+ 26.1%), tyrosine (+ 11.6%), total BCAA (+ 26.8%), and total EAA (+ 15.6%) maximum concentrations (Table 6).

**Table 6:** Individual amino acids, total BCAA, total EAA, and total amino acid maximum concentration (Cmax)

Cmax [μmol/L]							
Amino acid	Protein plus probiotic		Protein		Protein plus probiotic vs. protein		
	Mean	SD	Mean	SD	*p* value (*t* test)	Difference (%)	Effect size (d)
Arginine	102.4	30.9	106.6	42.9	0.556	− 3.9	− 0.11
Glutamine	601.7	131.8	599.9	131.2	0.974	0.3	0.01
Citrulline	26.4	9.7	27.4	10.5	0.580	− 3.6	− 0.10
Serine	128.3	28.4	130.4	47.5	0.860	− 1.6	− 0.05
Asparagine	101.3	22.6	96.1	31.0	0.602	5.5	0.19
Glycine	228.3	56.7	237.3	66.5	0.625	− 3.8	− 0.15
Threonine	161.9	30.5	159.2	51.0	0.861	1.7	0.06
Alanine	414.6	148.2	389.6	135.3	0.418	6.4	0.18
Ornithine	72.8	23.4	72.0	23.7	0.881	1.2	0.04
Methionine	23.2	8.9	19.9	6.3	0.008*	16.3	0.42
Proline	318.7	129.1	323.0	176.2	0.905	− 1.3	− 0.03
Lysine	276.0	45.9	266.0	70.5	0.627	3.7	0.17
Aspartic acid	7.3	3.6	5.9	3.6	0.208	22.7	0.37
Histidine	110.0	59.1	73.7	24.5	0.048*	49.2	0.80
Valine	490.5	174.5	393.4	94.2	0.034*	24.7	0.69
Glutamic acid	50.4	24.3	51.9	22.5	0.740	−3.0	−0.07
Tryptophan	90.8	24.7	88.7	19.7	0.796	2.4	0.10
Leucine	200.7	80.3	160.3	71.6	0.043*	25.2	0.53
Phenylalanine	57.6	16.8	54.8	18.9	0.252	5.2	0.16
Isoleucine	149.4	46.8	118.4	41.6	0.020*	26.1	0.70
Cysteine	19.5	5.3	19.8	7.5	0.832	− 1.8	− 0.06
Tyrosine	70.6	23.2	63.3	22.6	0.014*	11.6	0.32
Total BCAA	833.8	237.5	657.8	192.6	0.009*	26.8	0.81
Total EAA	1512.6	315.7	1308.2	301.3	0.022*	15.6	0.66
Total amino acids	3566.6	586.2	3389.4	818.6	0.371	5.2	0.25

Probiotic administration did not alter the time to reach the maximum concentration (Table 7).

**Table 7:** Individual amino acids, total BCAA, total EAA, and total amino acid time to maximum concentration (Tmax)

Tmax [min]							
Amino acid	Protein plus probiotic		Protein		Protein plus probiotic vs. protein		
	Mean	SD	Mean	SD	*p* value (*t* test)	Difference (%)	Effect size (d)
Arginine	58.0	41.6	40.0	14.6	0.057	45.0	0.58
Glutamine	54.0	41.2	60.0	56.7	0.748	− 10.0	− 0.12
Citrulline	56.0	46.6	48.0	49.2	0.546	16.7	0.17
Serine	52.0	40.0	40.0	29.3	0.271	30.0	0.34
Asparagine	58.0	43.1	46.0	33.8	0.395	26.1	0.31
Glycine	70.0	55.2	52.0	56.1	0.398	34.6	0.32
Threonine	62.0	51.3	50.0	40.4	0.458	24.0	0.26
Alanine	78.0	54.1	56.0	49.3	0.294	39.3	0.43
Ornithine	72.0	45.1	52.0	33.0	0.096	38.5	0.51
Methionine	48.0	42.1	38.0	13.7	0.388	26.3	0.32
Proline	66.0	53.4	56.0	54.2	0.632	17.9	0.19
Lysine	50.0	40.4	54.0	44.2	0.784	− 7.4	− 0.09
Aspartic acid	46.0	35.6	50.0	43.4	0.774	− 8.0	− 0.10
Histidine	62.0	51.3	44.0	43.7	0.246	40.9	0.38
Valine	60.0	40.9	58.0	53.7	0.914	3.4	0.04
Glutamic acid	42.0	37.3	74.0	69.8	0.088	− 43.2	− 0.57
Tryptophan	52.0	43.1	72.0	54.1	0.191	− 27.8	− 0.41
Leucine	60.0	40.9	44.0	15.5	0.120	36.4	0.52
Phenylalanine	52.0	24.0	42.0	15.2	0.096	23.8	0.50
Isoleucine	52.0	38.4	44.0	15.5	0.413	18.2	0.27
Cysteine	54.0	45.6	72.0	58.7	0.447	− 25.0	− 0.34
Tyrosine	58.0	36.7	46.0	15.5	0.233	26.1	0.43
Total BCAA	66.0	42.7	54.0	39.6	0.458	22.2	0.29
Total EAA	60.0	40.9	54.0	39.6	0.689	11.1	0.15
Total amino acids	58.0	43.1	60.0	52.0	0.910	− 3.3	− 0.04

## Discussion

Growing transition to more plant-based, whole-food, sustainable diets raised questions about the protein adequacy of vegetarian and vegan diets. In addition, a reduction of animal protein intake, in particular meat consumption, has recently become more prevalent in Western countries. Protein intake is usually the highest in meat-eaters, followed by fish-eaters, lacto-ovo vegetarians, and vegans [16]. Insufficient protein intake from vegetarian diets may occur if the diets are low in protein-rich foods such as legumes, nuts, or seeds; however, there is currently no evidence that a balanced vegetarian diet provides inadequate total protein intakes [16]. Plant proteins differ in digestibility and amino acid composition from animal proteins. Specifically, plant proteins such as pea or rice are lower in leucine and total BCAA content, amino acids that have been linked to muscle health by activating MPS [17]. In contrast to omnivores, people following a vegetarian diet have lower circulating serum BCAA concentrations, which have been linked to lower dietary intake of BCAAs and also to changes in the gut microbiota composition resulting in upregulation of the gut microbial pathway for the degradation of BCAAs [18].

Probiotics function predominately in the large intestine. There is currently no evidence that amino acids liberated from bacterial fermentation in the large intestine alter plasma amino acid concentrations of the host; however, certain probiotic strains have proteolytic properties and have been linked to an increased production of digestive enzymes and subsequently improved host protein utilization [12]. Thus, the purpose of this study was to investigate the individual and potential additive or synergistic effects of *L. paracasei* LP-DG® and/or *L. paracasei* LPC-S01 on the in vitro digestion of different plant protein sources, in conjunction with a human study investigating post-prandial changes in blood amino acids in response to a 20 g protein bolus, with or without the probiotic. The primary findings of the in vitro data showed increased proteolysis and a synergistic effect with the combination of *L. paracasei* LP-DG® and *L. paracasei* LPC-S01 (+ 6.5% at 220 nm; + 5.6% at 280 nm) compared with either strain alone for pea protein. Similar to the in vitro data, results from the human clinical trial showed increases in AUC for total BCAA (+ 22.8%) and total EAA (+ 16.0%) concentrations after 2 weeks of probiotic supplementation. Moreover, increases in leucine (+ 23.3%), isoleucine (+ 26.0%), valine (+ 21.5%), and total EAA (+ 16.0%) absorption suggest a potential for administering *L. paracasei* LP-DG® and *L. paracasei* LPC-S01 for optimization of amino acid absorption following pea protein consumption. Taken together, these data illustrate that this could be a strategy to enhance absorption levels of plant-based proteins and have implications on increasing protein utilization following the post-absorptive period in the human gut.

For building muscle mass and for maintaining muscle mass through a positive muscle protein balance, an overall daily protein intake in the range of 1.4–2.0 g protein/kg body weight/day (g/kg/d) is sufficient for most exercising individuals, and acute protein doses should strive to contain 1700 to 3000 mg of leucine, in addition to a balanced array of 10–12 g of EAAs [14]. EAAs play a critical role for achieving maximal rates of MPS making protein sources that are rich in EAAs the preferred sources of protein [14]. Amino acid analysis of the study material confirmed the relatively low leucine content (approx. 8%) of pea protein, in comparison with animal proteins such as whey protein, containing approx. 10–11% of leucine [19]. When compensating for the low leucine content by using large isonitrogenous doses (48–50 g of protein), rice [19] and pea protein [20] have been shown to be as effective as whey protein in increasing lean body mass and strength when combined with resistance exercise; however, the ability of standard protein doses (18–24 g of protein) has not been studied yet. Increasing leucine AUC concentration by 23% and total EAA concentrations by 16% by co-ingestion of probiotics helps to overcome the compositional shortcomings of plant proteins and could help to elevate BCAA levels in the blood to comparable levels of animal proteins, thereby potentially removing the need to increase the dose of plant proteins to achieve similar benefits of protein supplementation on muscle health. In addition to increasing the effects of resistance training on MPS, protein rich diets are used for weight management and weight loss during calorie-restricted diets. The total amount of muscle mass is the sum of MPS and muscle protein breakdown (MPB). A net positive muscle protein balance can be achieved if MPS is greater than MPB [21]. During energy restriction, MPB increases, resulting in a loss of fat-free mass, particularly if rates of weight loss exceed 1.0% body mass loss per week [22]. Higher protein intakes (2.3–3.1 g/kg/d) may be needed to maximize the retention of lean body weight [14] and promotion of fat loss [23] in resistance trained subjects during hypocaloric periods. Plant proteins with the addition of probiotics might be more effective in maintaining muscle mass during calorie-restricted diets, due to the greater increase in hyperaminoacidemia, especially in plasma leucine levels that stimulate MPS [21].

Athletes have varying gut microbiota compositions that appear to reflect the activity level of the host in comparison with sedentary people, with the differences linked primarily to the volume of exercise and amount of protein consumption [24]. The probiotic strains used in this study have been shown to increase absorption of key nutrients of specific importance to athletes, such as amino acids from protein. In addition, probiotics offer various health benefits for active people [25], such as improving the integrity of the gut-barrier function [26], and the administration of selected anti-inflammatory probiotic strains has been linked to improved recovery from muscle-damaging exercise [27]. Immune health in athletes is compromised with excessive training load, psychological stress, disturbed sleep, and environmental extremes, all of which can contribute to an increased risk of respiratory tract infections [28]. Approximately 70% of the immune system is located in the gut, and probiotic supplementation has been shown to promote a healthy immune response [25]. Studies in athletes have shown to improve immune health by reducing the number of episodes, severity, and duration of upper respiratory tract infections following intense, prolonged exercise [29].

Pea and rice protein are both incomplete proteins [19]. While rice protein is low in lysine, pea protein is low in methionine and cysteine. Most commercial plant protein products combine pea and rice protein to create a plant protein blend matching the requirements of a complete protein, as rice protein has higher contents of methionine and cysteine, and pea protein is rich in lysine. The co-administration of probiotics with pea protein increased methionine AUC levels by 20% in our study, offering an alternative nutritional approach to overcome the low methionine content in pea protein. The increase in appearance in the blood of sulfur-containing amino acids might have an additional health benefit. While carbohydrate fermentation is linked to beneficial health effects on the host due to the generation of short-chain fatty acids, protein fermentation, which mainly occurs in the distal colon, has been linked to the production of metabolites such as sulfides, amines, or ammonia [30]. Thus, increased absorption of sulfur-containing amino acids by the host might reduce the formation of sulfides.

Limitation of our study was the lack of urine and fecal analysis to confirm improved protein retention. Plasma concentration of amino acids does not directly relate to muscle protein synthesis or breakdown, which would need to be determined by muscle biopsies. The in vitro analysis of “protein hydrolysis” under small intestinal conditions is not reflective of the in vivo response as probiotics function predominately in the large intestine. Future studies should investigate the effects on animal proteins.

## Conclusion

In conclusion, results from the present study indicate that co-administration of plant protein with a multi-strain probiotic can increase post-prandial changes in blood amino acids including EAAs and BCAAs, which are closely linked to optimization of muscle health. Probiotic supplementation can be an important nutritional strategy to overcome compositional shortcomings of plant proteins.
